# Band-Engineered α-Fe_2_O_3_@NiO P-N Heterojunction for Room-Temperature NH_3_ Detection and Real-Time Meat Spoilage Monitoring

**DOI:** 10.3390/nano15130987

**Published:** 2025-06-25

**Authors:** Mingjia Li, Gaoshan Zeng, Haoyue You, Ding Xi, Hui Huang, Xin Kou, Amjad Farid, Yongpeng Zhao

**Affiliations:** 1 College of Mechanical and Electrical Engineering, Sichuan Agricultural University, Ya’an 625000, China; 15828190324@163.com (M.L.); 202305625@stu.sicau.edu.cn (G.Z.); 13881154909@163.com (H.Y.); 18599952445@163.com (D.X.); 2 College of Resources, Sichuan Agricultural University, Chengdu 611130, China; 3 Plasma Processing of Electrode Materials Lab, Department of Physics, Government College University Faisalabad, Faisalabad 38000, Pakistan

**Keywords:** band-engineered, heterojunction structure, α-Fe_2_O_3_, room temperature, meat spoilage monitoring

## Abstract

Recent advancements in biomarker technology have revolutionized diagnostic and monitoring applications, yet their potential in food quality assessment remains largely untapped. Herein, we report a breakthrough in gas-sensitive nanocomposite engineering through the design of α-Fe_2_O_3_-NiO heterostructures synthesized via a single-step hydrothermal protocol. The introduction of NiO led to increased oxygen vacancies and active sites, thereby reducing the sensor’s operating temperature. Additionally, the P-N heterojunction structure promoted the redistribution of electrons and hole, thus enhancing its conductivity. The optimized sensor exhibited high sensitivity (75.5% at 100 ppm), fast response/recovery (20 s/92 s), and perfect selectivity for NH_3_ at room temperature. In the end, based on this sensor and combined with a Programmable Logic Controller (PLC), a rapid and nondestructive meat spoilage detection system was constructed to reflect the degree of spoilage of meat with the help of NH_3_ concentration, providing a valuable strategy for the application of biomarker detection in the food industry.

## 1. Introduction

Due to the global concern for ecosystem protection, the pursuit of high-quality development and livable environments has become a social consensus. Ammonia (NH_3_), a colorless gas with a strong, irritating odor, not only significantly reduces air quality but also disrupts the ecological balance by altering soil physicochemical properties and triggering aquatic eutrophication. Moreover, NH_3_ exhibits high corrosivity toward the human respiratory mucosa, where exposure to high concentrations of the gas may cause nasopharyngeal inflammation, pulmonary injury, and even neurological disorders or compromised consciousness [1,2,3,4]. The ubiquity of NH_3_ emission sources exacerbates these risks: industrial leaks during production/transport/storage, microbial decomposition of nitrogenous fertilizers and livestock manure in agriculture, and spoilage of protein-rich seafood [5,6,7,8]. Therefore, developing accurate, convenient, and cost-effective NH_3_ detection technologies has become critical for environmental sustainability and public health protection, with urgent applications in industrial safety, agricultural conservation, and food security [9,10].

Current traditional NH_3_ detection methods include chemical reagent detection, infrared absorption spectrometry (IRAS), and gas chromatography (GC) [11,12,13]. While chemical reagent detection offers operational simplicity, it is susceptible to environmental interference, discrete sampling limitations, and inability to support real-time monitoring. Infrared absorption spectrometry (IRAS) can detect NH_3_ relatively accurately; for example, Jian and colleagues used near-infrared absorption spectroscopy to study the H_2_O-broadening coefficient of the methane transition, whose line intensity can be used for the sensitive detection of CH_4_ [14]. This method clarifies the structure of molecules with the help of molecular absorption of infrared light; it is highly accurate but requires pure samples and expensive equipment. Gas chromatography, on the other hand, can separate various components of gases in complex gas environments to accurately detect a particular gas. For example, Sasaki and colleagues used gas chromatography–mass spectrometry to identify potent odor substances in roasted tea stems and assessed the characteristics of the roasted tea stems by comparing the concentrations of these substances [15]. Although this method can detect NH_3_, expensive equipment and complicated procedures limit its application in real-world environments. Intriguingly, gas sensors quantify target gases through redox-induced impedance changes in sensing materials, offering advantages in terms of cost efficiency, sensitivity, and rapid response for real-time monitoring. For instance, Fan and colleagues prepared ZnO-modified CNF gas-sensitive materials and fabricated gas-sensitive sensors [16]. The good response recovery speed, excellent flexibility, and stability make them potential candidates for practical applications. However, the synergistic optimization of existing sensors in terms of sensitivity, response recovery speed, and stability is still a challenge, and the core bottleneck lies in the design and modification of gas-sensitive materials [17,18,19,20,21,22,23,24].

α-Fe_2_O_3_, as a semiconducting metal oxide, demonstrates significant gas-sensing potential due to its thermal stability, broad-spectrum reactivity, and facile synthesis [25,26,27,28,29,30,31,32,33]. For example, Hung and colleagues synthesized nanospheres using aggregated α-Fe_2_O_3_ particles, and the gaps between the nanoparticles and the hollow structure of the material enhanced the specific surface area and showed good properties towards ethanol [34]. Tomic and colleagues synthesized α-Fe_2_O_3_ films with different morphologies using AACVD and achieved an excellent response to ethanol at 80 °C, with 90% humidity tolerance [35]. These studies collectively demonstrated the versatility of α-Fe_2_O_3_ in gas sensing applications. However, a critical limitation persists: the gas-sensitive materials made from α-Fe_2_O_3_ have higher operating temperatures (typically >150 °C) and are vulnerable to humidity, resulting in high energy consumption and limited environmental adaptability. How to improve its stability while lowering its operating temperature is yet to be thoroughly investigated. Meanwhile, the surface chemical state, energy band structure, and lattice defects of metal oxides can be modulated by compositing with other materials to optimize their interaction with the target gas and reduce the operating temperature [36,37,38,39,40,41,42]. Precious metals, on the other hand, are often used as the activation centers through spillover effects, where dissociated oxygen species migrate to the metal oxide surface, lowering the activation energy for gas adsorption. For example, Zhu and his colleagues made the WO_3_/Au/SnO_2_ composite film by radio-frequency magnetron sputtering and ion beam sputtering, and this structure had perfect performance for acetone (21.11 for 50 ppm), with a significant reduction in response/recovery time (5 s/150 s) [43]. In summary, the use of precious metals can optimize their electronic and crystal structures, but it inevitably increases the cost, and most of the studies were able to improve the sensitivity while lowering the operating temperature; however, simultaneously achieving high sensitivity, rapid response/recovery kinetics, and room-temperature operation remains elusive.

In response to the above-mentioned challenges, the primary approach in current research is to conduct multi-dimensional regulation of α-Fe_2_O_3_ through band-engineering strategies. For instance, Wang and his colleagues constructed Fe_2_O_3_/SnO_2_ heterojunction. By leveraging the energy-level differences between SnO_2_ and α-Fe_2_O_3_, they induced spontaneous electron transfer at the interface. In the end, the operating temperature was reduced to 200 °C. Moreover, it not only demonstrated high sensitivity to propanol but also exhibited an extremely fast response and recovery speed (3 s/4 s) [44]. Secondly, the Mirzaei team innovatively introduced narrow bandgap Co_3_O_4_ (1.3 eV) to construct a gradient band structure. Through the regulation of the surface oxygen vacancy concentration, they achieved continuous band bending, and the corresponding gas-sensing performance reached over eight times that of the original metal sensor [45]. Furthermore, Zhang and his team developed an InO_2_/Fe_2_O_3_ heterojunction. By doping In, lattice distortion occurred, increasing the content of oxygen vacancies. This led to a value of 31.7 for 100 ppm and a response/recovery time of 1 s/1 s [46]. These studies confirmed that by precisely designing the band-matching degree of heterojunctions, the adsorption energy could be optimized, and the reaction energy could be reduced, providing a new paradigm for breaking through the bottleneck of room-temperature gas sensing. Meanwhile, NiO is a common p-type semiconductor and serves as an ideal dopant for gas-sensing applications for its own abundance of oxygen vacancies and active sites. When forming p-n heterojunctions with n-type oxides, NiO modulates carrier concentrations and band structures, dramatically enhancing sensitivity and selectivity; for example, Guo and colleagues synthesized thin films of NiO/In_2_O_3_ using solvent–thermal and gas–liquid interfacial self-assembly, and the p-n heterojunction interface of NiO/In_2_O_3_ enhanced the detection of butanone (235.71 to 50 ppm) [47]. Meanwhile, NiO also exhibits intrinsic catalytic activity for specific gases (catalytic oxidation of formaldehyde), and the synergistic effect with doping can significantly reduce the operating temperature and enhance the selectivity; for example, Yang and colleagues used low-temperature vapor diffusion to cover PANI on NiO, which resulted in excellent gas-sensitive performance at room temperature (12.21–100 ppm) [48]. In addition to this, the energy band structure (~3.6–4.0 eV) and the figure of merit of NiO can be further finely tuned by doping (Co, Fe, Cu) or using a special design to optimize the adsorption energy and conductivity response to specific gases. For example, Ezema and colleagues prepared GO/NiO composites using the solution gel technique and found that the bandgap value of the material was reduced to 1.86 ev after compositing; the carrier concentration also rose and the gas-sensitive performance increased [49]. In summary, NiO showed an irreplaceable role in enhancing sensitivity, selectivity, response speed, stability, and reducing power consumption, and was an ideal dopant for designing high-performance, low-power gas sensors.

NH_3_ sensors have shown significant potential for real-time monitoring of meat spoilage, based on the biochemical process of meat spoilage: spoilage microorganisms break down proteins to produce a wide range of biogenic amines, which are further degraded in subsequent metabolism, releasing the characteristic volatile ammonia (NH_3_) gas [50,51,52]. Therefore, changes in NH_3_ concentration can be used as a key volatile marker to characterize the degree of protein degradation and early meat spoilage. The development of gas-sensitive materials with high selectivity, high sensitivity, and excellent immunity to interferences is the key to realizing this application. For example, the TiO_2_/Ti_3_C_2_T_x_ composites synthesized by Zhang and colleagues using the hydrothermal method have a unique heterostructure, which not only endows the materials with good thermal stability but also improves the response to NH_3_ significantly [53]. Based on this sensor technology, a prototype of an early warning system for real-time assessment of the freshness of meat (fish) was successfully constructed by setting reasonable NH_3_ concentration thresholds (1 ppm for freshness and 5 ppm for significant spoilage). This demonstrates the feasibility of using NH_3_ sensors to monitor meat spoilage. However, establishing refined multiparameter correlations between NH_3_ levels and spoilage progression requires further investigation.

Inspired by the above studies, α-Fe_2_O_3_, which has excellent detection potential for NH_3_, was selected as the sensing substrate for gas sensors in this work, and NiO nanoparticles were introduced by a one-step hydrothermal method. The introduction of NiO enhanced the response/recovery ability (20 s/92 s) and the sensitivity of NH_3_ sensing (100 ppm for 75.5%) at room temperature, leading to efficient detection of NH_3_. In addition, based on the trend of increasing concern about meat food spoilage, a room temperature meat spoilage detection system was constructed on the basis of the above NiO room temperature gas-sensitive sensors with a Programmable Logic Controller as the control center, providing a low-cost, high-efficiency, and high-precision solution for meat spoilage detection.

## 2. Experimental Methods

### 2.1. Materials

All chemicals were analytical grade reagents and used without further purification. Both nickel nitrate hexahydrate (Ni(NO_3_)_2_·6H_2_O, 98.0% purity) and ferric nitrate nonahydrate (Fe(NO_3_)_3_·9H_2_O, 98.5% purity), which were employed as metal precursors, were supplied by Chron Chemicals Co., Ltd. (Chengdu, China). Urea (CO(NH_2_)_2_, 99.0% purity) was obtained from Jinshan Chemical Co., Ltd. (Chengdu, China).

### 2.2. Synthesis Process

**Figure 1:**
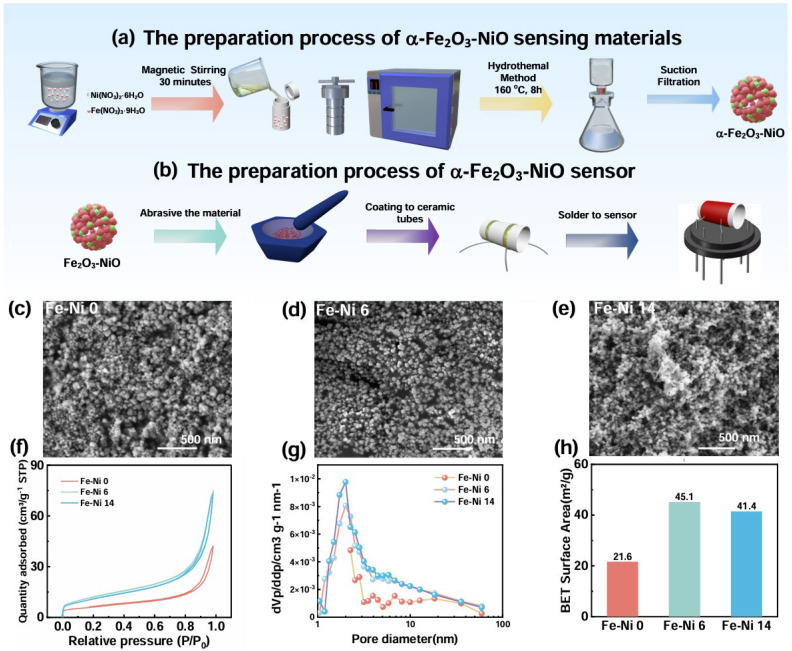
(**a**) Preparation process for α-Fe_2_O_3_-NiO; (**b**) Preparation process for sensors; SEM images of (**c**) Fe-Ni 0; (**d**) Fe-Ni 6; (**e**) Fe-Ni 14; (**f**) the N_2_ adsorption–desorption Isotherms of Fe-Ni 0, Fe-Ni 6, and Fe-Ni 14; (**g**) the pore size distribution in Fe-Ni 0, Fe-Ni 6, and Fe-Ni 14; (**h**) specific surface areas of Fe-Ni 0, Fe-Ni 6, and Fe-Ni 14.

In this study, stoichiometric combinations of nickel nitrate hexahydrate Ni(NO_3_)_2_·6H_2_O and ferric nitrate nonahydrate Fe(NO_3_)_3_·9H_2_O were prepared with varying Fe/Ni molar ratios (100:0, 98:2, 94:6, 90:10, 86:14) in 70 mL deionized water, followed by addition of 1 g urea (CO(NH_2_)_2_). The Fe(NO_3_)_3_·9H_2_O (404 mg) was combined with 0 mg, 6 mg, 18.6 mg, 32.3 mg, and 47.4 mg of Ni(NO_3_)_2_·6H_2_O. The homogeneous solution obtained through magnetic stirring (15 min) was subsequently subjected to solvothermal treatment in a 100 mL polytetrafluoroethylene hydrothermal reactor at 160 °C for 8 h and then washed four times with a mixture of deionized water and anhydrous ethanol. The synthesized α-Fe_2_O_3_@0%NiO, α-Fe_2_O_3_@2%NiO, α-Fe_2_O_3_ @6%NiO, α-Fe_2_O_3_ @10%NiO, and α-Fe_2_O_3_ @14%NiO samples were named Fe-Ni 0, Fe-Ni 2, Fe-Ni 6, Fe-Ni 10, and Fe-Ni 14. The specific process is shown in Figure 1.

### 2.3. Characterization

The structural and functional properties of the material were comprehensively characterized through multiple analytical techniques. Morphological features were investigated using field emission scanning electron microscopy (FE-SEM, Nova NanoSEM450, Brno, Czech Republic) coupled with transmission electron microscopy (TEM, JEOL JEM-2100, Akishima, Tokyo, Japan) for nanoscale visualization. Crystalline phase identification was conducted via X-ray diffraction (XRD, Rigaku innovative lab, Akishima, Tokyo, Japan) with 2θ scanning from 5° to 80°. Surface characteristics were quantified through Brunauer–Emmett–Teller (BET) surface area analysis using a Micromeritics TriStar II 3020 system (Norcross, GA, USA). The chemical composition was investigated X-ray photoelectron spectroscopy (XPS, ThermoFisher Nexsa G2, Thermo Fisher Scientific in East Grinstead, UK), it uses monochromatic light elements with a resolution of less than 0.5 ev. while electrical performance was assessed through current-voltage (I–V) measurements recorded with a Keithley 2450 source meter (Cleveland, OH, USA).

### 2.4. Gas Sensing Testing

The gas sensing performance of the synthesized materials was systematically evaluated using the following methodology. As illustrated in Figure 1, all samples were uniformly ground and deposited onto ceramic tubes that were subsequently soldered to a sensor base. Characterization was conducted through a gas sensing analysis system (WS-30b, Weisheng Company, Zhengzhou, China). During testing, the electrical resistance of representative sensors was continuously monitored, with R_a_ denoting the baseline resistance in air and R_g_ representing the resistance upon exposure to target analytes. Sensor response (S) was quantitatively determined through the formula S=(Ra − Rg)/Ra×100%. Operational dynamics were further characterized by two temporal parameters: response time (duration to attain 90% of maximum resistance variation during analyte exposure) and recovery time (period required to regain 90% of baseline resistance after analyte removal), which collectively reflect the sensor’s performance.

To assess their ability to detect NH_3_, sensing experiments were conducted for each sample. During the testing process, the gas testing system performed all the tests. The formula for the concentration of NH_3_ is as follows:

(1) $$\frac{mg}{m^{3}}=\frac{M}{22.4}\times \frac{273.15}{273.15+T}\times \frac{P}{101325}ppm$$

The equation parameters are defined as follows: M, the target gas’s molar mass; P, the standard atmospheric pressure; and T, the temperature. We performed the gas sensing test at 20 °C; 100 ppm of NH_3_ corresponds to 70.7 mg/m^3^.

(2) $$V_{x}=\frac{V\;*\;C\;*\;M}{22.4\;*\;d\;*\;p}\;*\;10^{-9}*\frac{273+T_{r}}{273+T_{b}}$$

In the above gas distribution formula, V_x_ is the required volume of ammonia solution, V is the test chamber solvent in milliliters, C is the liquid vapor concentration in parts per million, M is the molecular weight of the liquid in grams (g), d is the specific gravity of the liquid in grams per cubic centimeter (g/cm^3^), p is the purity of the liquid, Ta is the temperature at room temperature in degrees Celsius (°C). The ammonia solution was adopted from Cologne (35), the specific gravity of the liquid is 0.91, and the purity of the liquid is 25%. The Weisheng Gas Sensing System (WS-30, Zhengzhou, China) has a chamber volume of 18,000 mL; thus, the 100 ppm ammonia we tested was equivalent to 12 µL using the above formula.

## 3. Results and Discussion

### 3.1. Morphology and Structural Characterizations

The α-Fe_2_O_3_-NiO-sensing materials were synthesized via a simple hydrothermal method illustrated in Figure 1a. Firstly, stoichiometric ratios of Fe_2_O_3_ and NiO precursors were precisely weighed and homogenized using blenders (15 min). The mixed precursors were then subjected to solvothermal treatment in a 100 mL polytetrafluoroethylene hydrothermal reactor preserved at 160 °C under atmospheric pressure for 8 h. Subsequently, the resultant precipitate was collected through vacuum filtration and thoroughly washed four times. The purified product was finally obtained after drying at 60 °C for 2 h. As depicted in Figure 1b, the obtained nanocomposite powder was dispersed in alcohol using an ultrasound machine to achieve a homogeneous colloidal suspension, followed by uniform coating on a ceramic tube and integration with a heating wire via soldering.

The microscopic morphology of all composites was first observed and analyzed by SEM. Due to the addition of urea in the hydrothermal experiment, pyrolysis reaction occurred at high temperature, which produced NH_3_ and carbon dioxide, triggering a complex chemical reaction that affected the formation and crystal structure of α-Fe_2_O_3_, leading to inconsistent crystal growth direction. Figure 1c shows the smooth, uniform particles. As shown in Figure 1c–e, the growth of α-Fe_2_O_3_ was inhibited as the NiO content increased, and the overall morphology tended to consist of more refined nanoparticles, while also introducing more pores due to the interfacial energy difference. When composited with an excessive amount of NiO, it combines with α-Fe_2_O_3_ particles on the surface of the excessive chemical adsorption or surface coordination and agglomeration, ultimately reducing the specific surface area through pore obstruction. Notably, the Fe-Ni 6 particles shown in Figure 1 are uniformly distributed and have a porous-type structure, suggesting that the addition of NiO in this amount contributed to the growth of the sensor material, while numerous pores were formed at the interface, promoting gas diffusion and adsorption.

Additionally, N_2_ physisorption characterization revealed type IV isotherms (Figure 1f) and 2.01 nm mesopores (Figure 1g) in Fe-Ni sensors, with specific surface areas increasing from 21.6 m^2^/g (Fe-Ni 0) to 45.1 m^2^/g (Fe-Ni 6), and then decreasing to 41.4 m^2^/g (Fe-Ni 14) (Figure 1h), facilitating electron transport and adsorption of NH_3_ molecules. This textural evolution facilitates electron transport and NH_3_ adsorption, with porosity optimization consistent with SEM observations.

**Figure 2:**
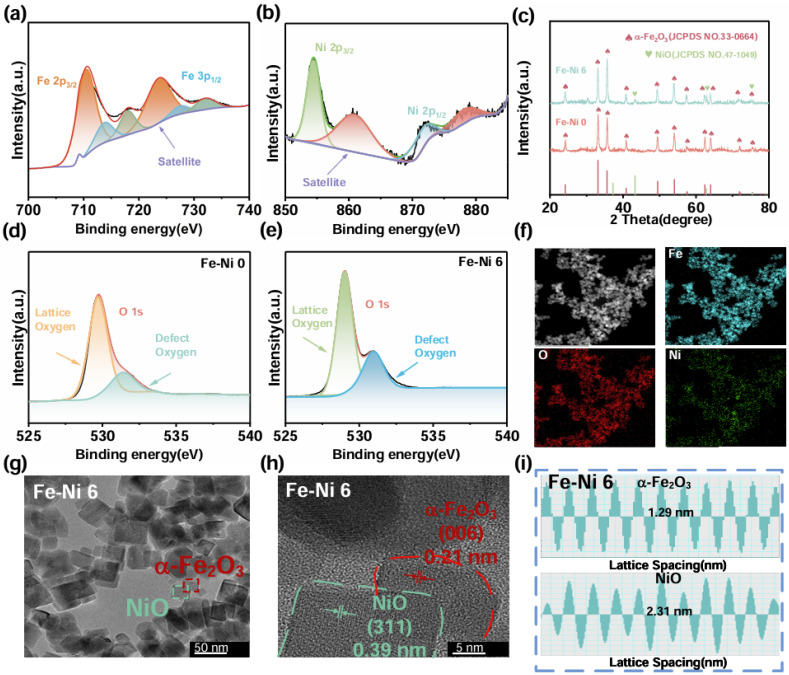
(**a**) XPS full spectrum of Fe-Ni 0; (**b**) XPS Ni 2p fine spectrum of Fe-Ni 6; (**c**) XRD diffraction of different samples; (**d**) XPS O 1s fine spectrum of Fe-Ni 0; (**e**) XPS O 1s fine spectrum of Fe-Ni 6; (**f**) EDS mapping of Fe-Ni 6; (**g**) LRTEM; (**h**) HRTEM; (**i**) Inverse Fast Fourier Transform.

XPS characterization elucidated the chemical states and surface defects in the composite system. Figure 2 and Figure S1 show the corresponding spectra. Figure S1a,b show the XPS spectra of Fe-Ni 0 and Fe-Ni 6, respectively. In both samples, the characteristic peaks of Fe and O are clearly visible, while the characteristic peaks of the element Ni are present in Fe-Ni 6, consistent with the SEM and EDS results. Due to the introduction of additional defect sites after compositing NiO on the α-Fe_2_O_3_ surface, it was able to adsorb gas molecules and increase the adsorption capacity of the sensing material to the target molecules. As a result, the peak areas of the defective oxygen peaks, as shown in Figure 2d,e, were significantly increased. The high-resolution Fe-2p spectrum in Figure 2a shows characteristic peaks at 723.70 eV and 710.26 eV for Fe 2p1/2 and Fe 2p3/2 [54,55,56], which correspond to the Fe^3+^ values in the standard database. The high-resolution Ni 2p spectrum shown in Figure 2b shows characteristic peaks at 873.80 eV and 855.80 eV, which belong to Ni 2p1/2 and Ni 2p3/2 [57,58,59], respectively, corresponding to the Ni^2+^ value in the standard database. These results further confirmed the successful compositing of α-Fe_2_O_3_ with NiO.

Critically, the O 1s spectra (Figure 2d,e) revealed significant surface modifications upon NiO compositing: lattice oxygen (529.7 eV), oxygen vacancies/surface defects (532.0 eV) [60,61,62]. Notably, the relative area of the O defect component increased substantially from Fe-Ni 0 to Fe-Ni 6. This pronounced increase in oxygen vacancy concentration was attributed to the interfacial effects arising from the heterojunction formation between α-Fe_2_O_3_ and NiO, potentially involving lattice strain and charge redistribution. These oxygen vacancies played a pivotal role in enhancing the gas sensing performance. They served as preferential adsorption sites for both atmospheric oxygen (facilitating its dissociation into reactive ionic species O^−^/O_2_^−^) and target gas molecules (NH_3_). Furthermore, the defect states associated with Vo within the bandgap promote efficient charge exchange during gas adsorption/desorption processes. Therefore, the synergistic effect of the α-Fe_2_O_3_/NiO heterojunction and the significantly increased surface oxygen vacancies, as directly evidenced by XPS, underpinned the enhanced adsorption capacity and sensing response observed in the composite material.

Next, the image structure of the resulting samples was determined by XRD diffraction tests. Figure 2c shows that all the diffraction peaks of Fe-Ni 0 belong to α-Fe_2_O_3_ (JCPDF: 330664). The characteristic peaks at 24.01°, 33.07°, 35.52°, 40.76°, 49.30°, 53.91°, 62.33°, 63.92°, 69.52°, 71.8°, and 75.42° belong to α-Fe_2_O_3_ at (012), (104), (110), (113), (024), (116), (018), (214), (300), (208), (119), and (220) facets. Each characteristic peak corresponds to the triangular corundum structure of α-Fe_2_O_3_ (JCPDF: 33-0664) [63], and no other phases were detected. The good crystallinity of NiO, on the other hand, made it possible to detect the diffraction peaks of NiO (JCPDF: 47-1049) [64]. Even though its content was very low, it still showed that the characteristic peaks at 43.29°, 62.91°, and 75.44° belong to the (200), (220), and (311) faces of NiO [65]. The results of elemental analysis by EDS (Figure 2f) showed that Fe, O, and Ni are uniformly distributed in the sensing material, corresponding with the XPS results, indicating the chemical composition’s consistency; however, due to the low content of composited Ni, the content of Ni, although uniformly distributed, is significantly less than Fe and O.

Furthermore, the internal structure of the Fe-Ni 6 sample was analyzed by TEM. The low-magnitude image (Figure 2g) exhibited mesoporous morphology with surface defects, corroborating SEM observations. The high-resolution TEM is shown in Figure 2h, and Figure 2i shows that the corresponding fast inverse Fourier transform images and two different dot-matrix striped with characteristic intervals of 0.21 nm and 0.39 nm were observed in the IFFT maps of the Fe-Ni 6 samples, which correspond to the surfaces of the α-Fe_2_O_3_ (006) and NiO (311) crystals, respectively. Figure 2h shows that α-Fe_2_O_3_ and NiO formed a heterojunction at the contact surface. The lattice mismatch and electronic energy level difference in the heterojunction region might result in an energy band shift and electron scattering phenomenon, and the energy band structure formed by two different metal oxides would form an energy gradient, prompting the flow of electrons from high-energy to low-energy, thus facilitating electron transport between the materials. At the same time, the formation of heterojunctions enhanced the electron transport properties of the materials because it increased the diffusion length of the carriers, which in turn increased the transport rate of the carriers, and hence the response recovery speed.

### 3.2. Gas Sensing Performance

**Figure 3:**
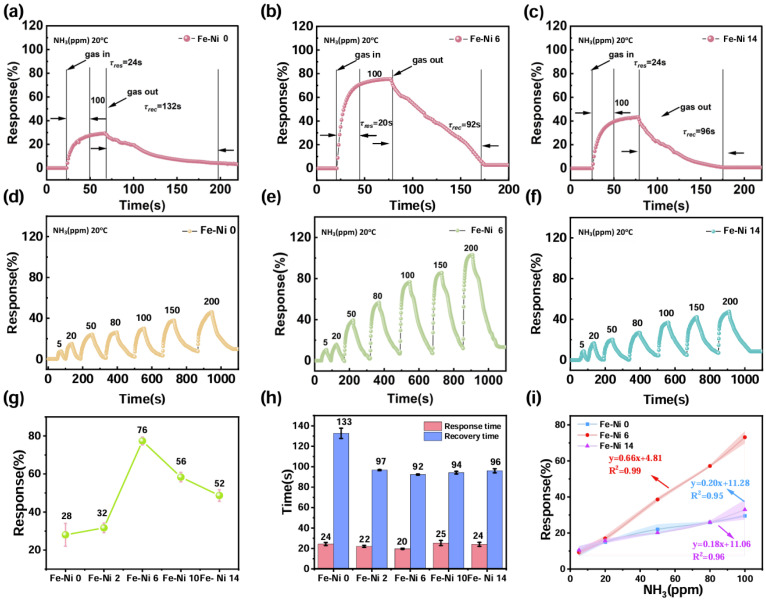
Response/recovery characteristics of (**a**) Fe-Ni 0, (**b**) Fe-Ni 6, and (**c**) Fe-Ni 14. Response curves of different concentrations of (**d**) Fe-Ni 0, (**e**) Fe-Ni 6, and (**f**) Fe-Ni 14. (**g**) The response values of different samples. (**h**) Response and recovery times of different samples. (**i**) Linear relationships of the responses of different concentrations.

Sensor sensitivity, a pivotal performance metric for metal oxide detectors, was quantitatively assessed through NH_3_ response dynamics. Figure 3a–c and Figure S3 show the response image of Fe-Ni 6 for 100 ppm NH_3_ at 20 °C, which not only reflects the sensitivity but also the response/recovery time. All the data are summarized in Figure 3g,h. The response value of Fe-Ni 6 at room temperature for 100 ppm NH_3_ is 75.5%, the highest of all the sensors; the pure sample only exhibited a response value of 28.3%. This performance leap directly correlated with the optimized textural properties evidenced by SEM. NiO incorporation increased the specific surface area from 21.6 to 45.1 m^2^/g while establishing mesoporous networks (2.01 nm average pore size). However, excessive NiO loading induced particle agglomeration, reducing active surface area by 8.2% (41.4 m^2^/g for Fe-Ni 10) through pore blockage. The composition-dependent sensitivity profile confirmed NiO’s dual role as both a surface modifier (enhancing gas accessibility) and electronic sensitizer (facilitating charge transfer), ultimately positioning Fe-Ni 6 as an optimal candidate for real-time NH_3_ monitoring.

Figure 3d–f show the response curves of Fe-Ni 0, Fe-Ni 6, and Fe-Ni 14 at different concentrations; it is clear that all three gas sensors have high responses at high concentrations in Figure 3e, which possesses a higher response value and a wider detection range than Fe-Ni 0 and Fe-Ni 14. Not only did Fe-Ni 6 have the highest response values (Figure 3g), but it also had the fastest response/recovery times (Figure 3h). Analyzing the linear relationships showed that the Fe-Ni 6 sensor had a highly linear relationship (R^2^ = 0.99) in the detection range of 5–100 ppm (Figure S4), while other samples showed a weaker linear relationship (R^2^ = 0.96 and R^2^ = 0.95), with the shaded area representing its three-fold confidence interval. The near-ideal linear relationship between NH_3_ concentration and response value in this detection range provides a reliable basis for quantitative analysis of the sensor in applications and the reliability of the system measurements. Finally, we calculated the detection limit of Fe-Ni 6 to be 7.95 ppm, making it a more sensitive gas-sensitive material based on the linear range of Fe-Ni 6 in the concentration gradient according to $\frac{3(NH_{3)}}{(R-R_{0})/\alpha }$.

**Figure 4:**
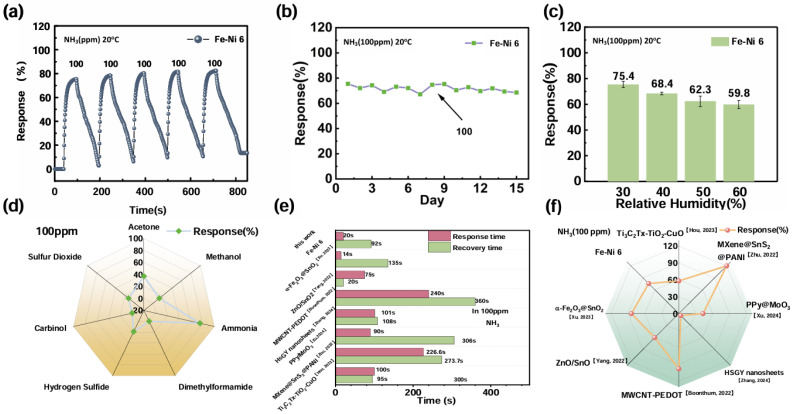
(**a**) Cyclic stability of Fe-Ni 6; (**b**) long-term stability; (**c**) stable responsiveness under different humidity conditions; (**d**) gas selectivity by Fe-Ni 6; (**e**) comparison of response/recovery times; (**f**) comparison of sensitivity [66,67,68,69,70,71,72].

The stability in different environments was a strong guarantee for application in practical testing. Therefore, we tested the Fe-Ni 6 sensor at room temperature under different humidity conditions and under 100 ppm cycling. Figure 4a,b show the respective results. In general, Fe-Ni 6 showed excellent reliability and stability. The response of the sensor under 15 days of continuous operation is shown in Figure 4c, further proving that Fe-Ni 6 has strong stability. A variety of gases are generated during the process of meat spoilage. In order to exclude the interference of other gases during meat spoilage detection, the gas sensor should have good selectivity. As shown in Figure 4d, among a series of interfering gases (acetone, methanol, formaldehyde, DMF, hydrogen sulfide, sulfur dioxide) tested, the Fe-Ni 6 sensor for NH_3_ had excellent selectivity and could eliminate interference by other gases. In summary, the Fe-Ni 6 sensor had excellent selectivity, high sensitivity, and perfect stability; thus, it can be used for practical testing.

Comparative analysis of NH_3_ sensor performance metrics (Figure 4e,f) positioned Fe-Ni 6 as having a slight advantage over recently reported metal oxide-based detectors, based not only on its sensitivity but also on its response/recovery speed. The accelerated surface reaction dynamics, facilitated by the optimized α-Fe_2_O_3_-NiO heterointerface, enable real-time monitoring capabilities for practical sensor deployment.

Finally, since the operating temperature caused changes in the carrier flow rate inside the material, we tested the response curves of Fe-Ni 6 at different operating temperatures; the data are summarized in Figure S5, which clearly shows that Fe-Ni 6 had the perfect performance at 20 °C. It was notable that the response/recovery time changed with the change in temperature. This proves the importance of the P-N heterojunction construction in a room temperature sensor. Therefore, without the need for an additional heating device, and thus reducing power consumption, Fe-Ni 6 had the perfect NH_3_-sensing performance.

### 3.3. Sensing Mechanism

**Figure 5:**
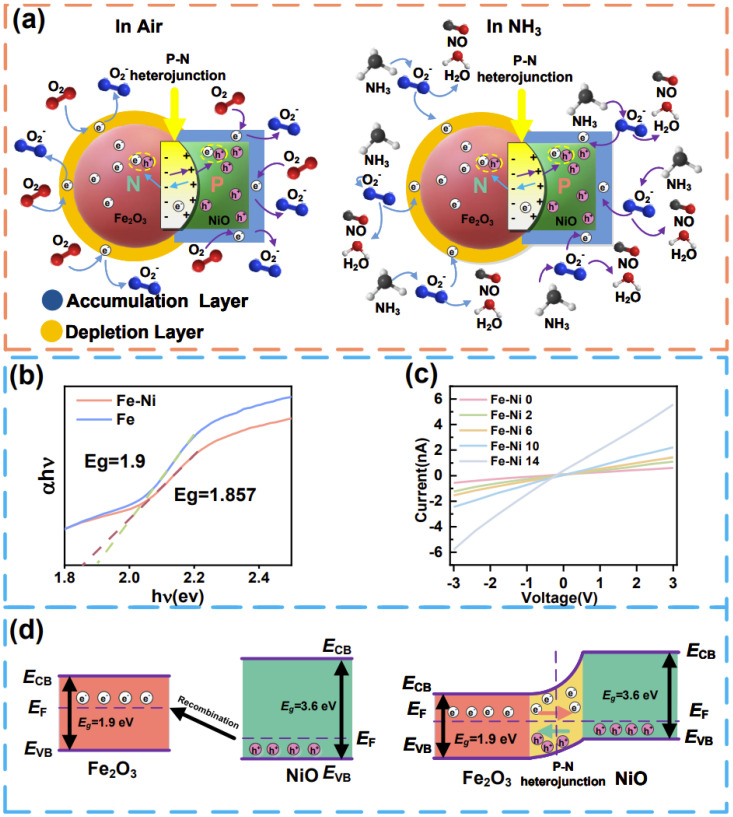
(**a**) Gas adsorption mechanism of α-Fe_2_O_3_-NiO; (**b**) the bandgap of α-Fe_2_O_3_-NiO; (**c**) I–V characteristic curves of different samples; (**d**) energy band structure of α-Fe_2_O_3_-NiO.

The mechanism of NH_3_ sensing in a typical α-Fe_2_O_3_-NiO p-n heterogeneous semiconductor involves a reaction between the adsorption of oxygen molecules on the target gas molecules [101] and the active site (Figure 5a). The sensing mechanism is initiated with oxygen chemisorption on the α-Fe_2_O_3_-NiO heterostructure, where adsorbed O_2_ molecules extract conduction band electrons to form activated oxygen species. Then, the NH_3_ molecules react with the oxygen ions to form NO and H_2_O when the sensor is exposed to NH_3_. In this reaction, the captured electrons are released back into the conduction band, thereby increasing the conductivity of the sensor and causing the resistance value of the sensor to decrease. The adsorption of oxygen molecules and the detection of NH_3_ gas can be expressed by the formulas in Equations (1) and (2).

(3) $$O_{2(ads)}+e^{-}\to {{O_{2}}^{-}}_{\left(ads\right)}$$

(4) $$4NH_{3}\left(g\right)+5{{O_{2}}^{-}}_{\left(ads\right)}\to 4NO\left(g\right)+6H_{2}O+5e^{-}$$

The superior gas-sensing capabilities of α-Fe_2_O_3_-NiO heterostructures originated from NiO-mediated dual optimization (the nanostructure optimization and electronic structure optimization), which collectively facilitated the release and transfer of electrons. Firstly, the strategic integration of NiO induced mesoporous architecture development (average pore size: 2.01 nm), achieving a 109% specific surface area enhancement that significantly improved gas–surface interaction efficiency through optimized molecular adsorption kinetics and reduced diffusion barriers. In addition, the porous structure also helped to increase the diffusion rate of gas molecules in the material and accelerated the response speed. Secondly, the introduction of NiO increased the content of oxygen vacancy, which provided more space for the adsorption of oxygen. The increase in adsorbed oxygen content provided more active sites and created a thicker electron depletion layer on the material surface by trapping conduction band electrons. The rise in resistance due to the electron depletion layer was the resistance R_a_ − R_g_ that changed when our sensors were exposed to the gas. As the sensitivity was defined as S=(Ra−Rg)/Ra×100%, a thicker electron depletion layer amplified a higher resistance Ra-Rg, thus leading to an increase in sensitivity.

The electronic structure of α-Fe_2_O_3_, a typical n-type semiconductor, was characterized by a significant energy separation between its valence and conduction bands. Its relatively large bandgap (2.1 eV) inherently resulted in poor electronic conductivity, as electrons required substantial energy to transition across this wide gap. In contrast, NiO exhibited p-type semiconducting behavior. The strategic compositing of NiO with α-Fe_2_O_3_ fundamentally modified the electronic structure and conductive properties of α-Fe_2_O_3_ (Figure 5d). While the formation of a p-n heterojunction typically establishes space-charge regions at the interface, increasing material resistance through carrier depletion, the incorporation of NiO simultaneously introduced effective acceptor doping into the α-Fe_2_O_3_ lattice. This doping effect dominantly elevated the majority carrier concentration, thereby reducing the overall electrical resistance. Experimental validation via UV-Vis spectroscopy and I–V measurements confirmed this mechanism: with increasing NiO content, the composite exhibited a narrowing bandgap, elevated carrier concentration, and reduced electrical resistance. These findings unambiguously demonstrated that the doping-induced enhancement in carrier concentration superseded the resistance-increasing effect of the space-charge region.

Therefore, the interfacial energy band alignment at the p-n junction generated an intrinsic electric field. This field actively promoted the drift motion of charge carriers during gas sensing events. The accelerated carrier drift velocity directly translates to faster sensor response kinetics. Consequently, the synergistic optimization—encompassing NiO-mediated special refinement (enhancing gas diffusion/adsorption) and electronic structure modulation (boosting carrier density and drift efficiency)—collectively elevated both the response magnitude and response/recovery speed.

### 3.4. The Meat Spoilage Test

Having developed a low-cost, high-performance gas-sensitive sensing material, we targeted its application in meat spoilage detection. Fresh beef, fish, and pork samples (5 g each) were prepared for controlled-environment storage and evaluated using gas sensors over five consecutive days. The results showed that the sensors had a significant response, which was capable of practical application.

**Figure 6:**
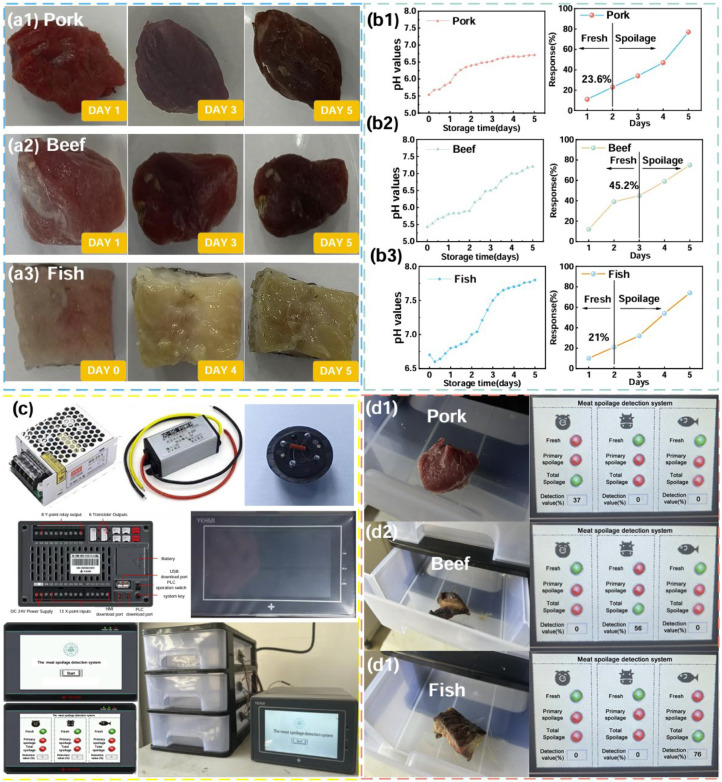
(**a1**–**a3**) The process of corruption of pork/beef/fish; (**b1**–**b3**) the PH value and sensor response criteria values for determining pork/beef/fish; (**c**) the meat corruption detection system; (**d1**–**d3**) actual test results of pork/beef/fish corruption detection system.

In order to calibrate the sensor values of the meat spoilage detection system, pork, beef, and fish samples underwent spoilage assessment over five days using two standard methods, namely the sensory assessment test method (GB 2707-2016 [73]) and PH determination. The three meats were selected and placed in a room temperature environment (20 °C ± 5 °C) with 45–60% relative humidity. Assessments occurred at 6 h intervals, with sensory evaluation involving volunteer grading of appearance, odor, and texture on a 5-point scale: 1 (spoiled), 2–3 (initial spoilage), and 4–5 (fresh). Concurrent pH measurements followed GB 5009.237-2016 protocols [74]. Sensor responses for all meats were recorded simultaneously to establish parameter correlations; the resultant data are shown in Figure 6(b1–b3) and Table S1. Immediately following this, we developed an algorithmic model between the degree of spoilage as judged by the PH value and the corresponding response values tested by the sensors. The judgement criteria are presented in Table S2. This calibration method combined several meat spoilage detection methods, fully considered the various changes in the meat spoilage process, and helped to improve the system’s ability to detect the degree of meat spoilage.

Subsequently, to check the practical performance of the fabricated α-Fe_2_O_3_-NiO sensor, we designed a meat spoilage detection system using a PLC as the control core, the prepared α-Fe_2_O_3_-NiO gas sensor as the detection module, and a 3D printed box as the housing (Figure 6c). The principle of the PLC control program is shown in Figure S6. During the testing process, meat samples were placed in the meat spoilage detection system; the system collected the gas released from the samples in real time through gas sensors and evaluated the degree of spoilage according to the pre-established correlation model. Finally, the assessment results were compared and analyzed with the actual situation to verify the performance of the system. When the NH_3_ concentration was within a certain threshold value of the system, the light indicating the response to the fresh condition turned green. Figure 6(d1–d3) show all the test results. In summary, this meat spoilage detection system showed high accuracy and reliability under the experimental conditions, providing a novel idea of detection technology in the field of food safety.

## 4. Conclusions

In summary, we demonstrated a cost-effective strategy for fabricating room-temperature NH_3_ sensors through precise band engineering of α-Fe_2_O_3_-NiO heterojunctions. By controlling the ratio, we enhanced both sensitivity and recovery performance. Compared to pure Fe sensors, Fe-Ni 6 sensors exhibited fast response/recovery capability (20/92 s) and high sensitivity (75.5%) toward NH_3_ at room temperature. The enhancement of the Fe-Ni 6 sensor can be attributed to the nanostructure optimization and electronic structure optimization brought by NiO. The compositing of NiO promoted the formation of porous structures and introduced crystal defects, thus increasing the specific surface area and providing more active sites. Additionally, it modified the conductive behavior of α-Fe_2_O_3_, accelerating NH_3_ adsorption and enabling room-temperature detection. Building on these findings, a fast and non-destructive meat spoilage detection system was designed using a Fe-Ni 6 sensor and PLC integrated control to reflect the degree of spoilage using the NH_3_ concentration produced during the meat spoiling process. This provides a novel strategy for the practical application of Biomarkers in the food field.

## Data Availability

Data are contained within the article and Supplementary Materials.

## Supplementary Materials

The following supporting information can be downloaded at: https://www.mdpi.com/article/10.3390/nano15130987/s1, Figure S1. XPS of (a) Fe-Ni 0; (b) Fe-Ni 6. Figure S2. The sensitivity UV–vis spectrum (a) Fe_2_O_3_; (b) Fe_2_O_3_-NiO. Figure S3. Response/recovery characteristics of (a) Fe-Ni 2; (b) Fe-Ni 10. Figure S4. Linear relationship of response to different concentrations. Figure S5. The different operating temperature of the response curve of Fe-Ni 6. Figure S6. Principle of PLC control program. Table S1. The results of the detection methods. Table S2. Threshold value of meat corruption detection system.
