# Supplementary material for: Band-Engineered α-Fe2O3@NiO P-N Heterojunction for Room-Temperature NH3 Detection and Real-Time Meat Spoilage Monitoring
Source: Nanomaterials (Basel). 2025 Jun 25;15(13):987. doi: 10.3390/nano15130987 (PMC12250746; doi:10.3390/nano15130987)
Supplement: Supplementary file 1 [file nanomaterials-15-00987-s001.zip › nanomaterials-3685198-supplementary.pdf]

Supporting Information

Band-Engineered α-Fe_2O_3@NiO P-N Heterojunction for Room-Temperature NH_3 Detection and Real-time Meat Spoilage Monitoring

Mingjia Li[1], Gaoshan Zeng[1], Haoyue You[1], Ding Xi[1], Hui Huang[1*], Xin Kou[2*], Amjad Farid[3*], Yongpeng Zhao[1*]

[1] *College of Mechanical and Electrical Engineering, Sichuan Agricultural University, Ya'an 625000, China*

[2] *College of Resources, Sichuan Agricultural University, Chengdu 611130, China*

[3]*Plasma Processing of Electrode Materials Lab, Department of Physics, Government College University Faisalabad, Faisalabad 38000, Pakistan*

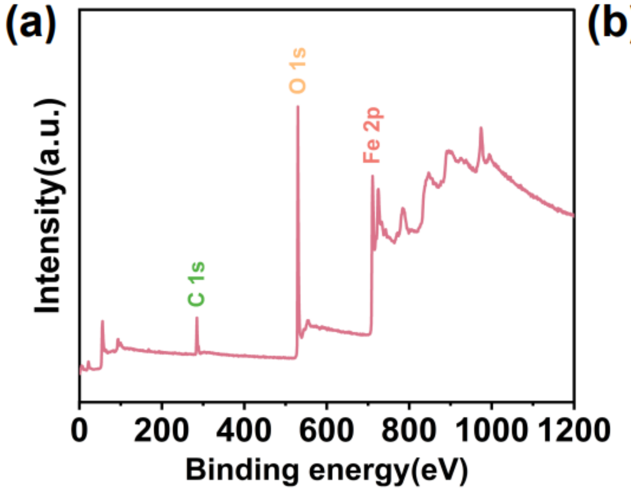

Figure S1. XPS of (a) Fe-Ni 0; (b) Fe-Ni 6.

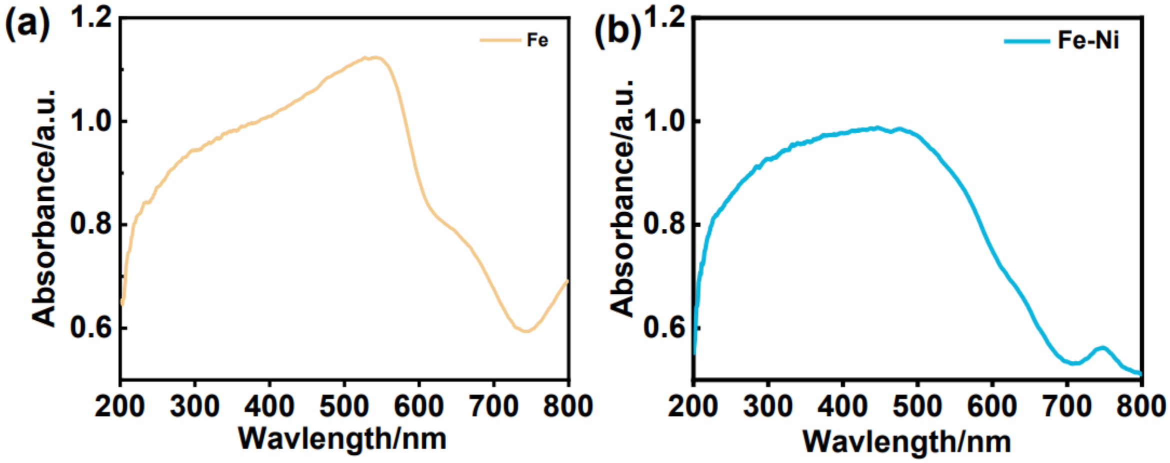

Figure S2. The sensitivity UV–vis spectrum (a) Fe_2O_3; (b) Fe_2O_3-NiO;

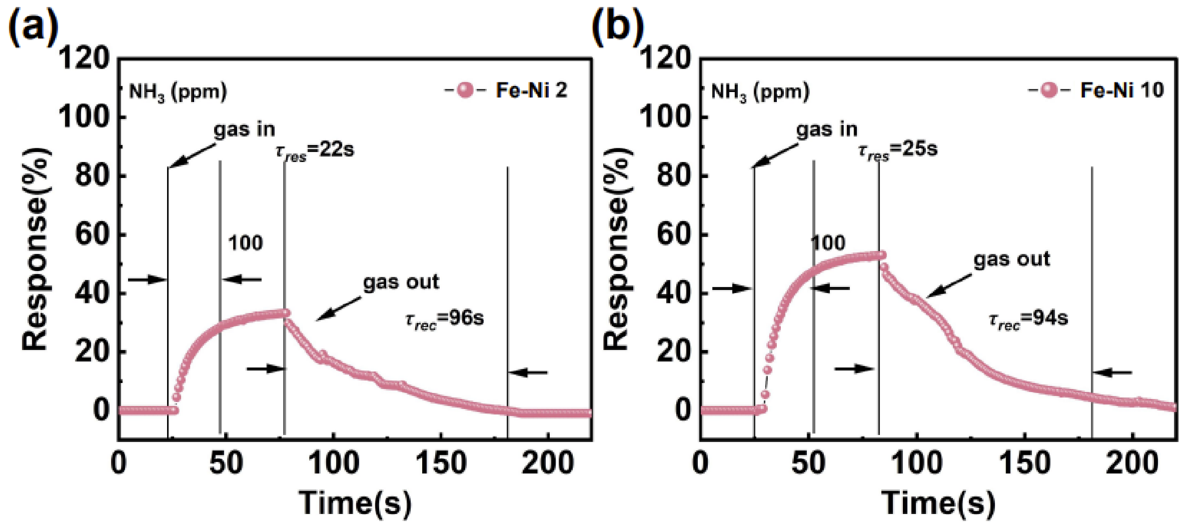

Figure S3. Response/recovery characteristics of (a) Fe-Ni 2; (b) Fe-Ni 10.

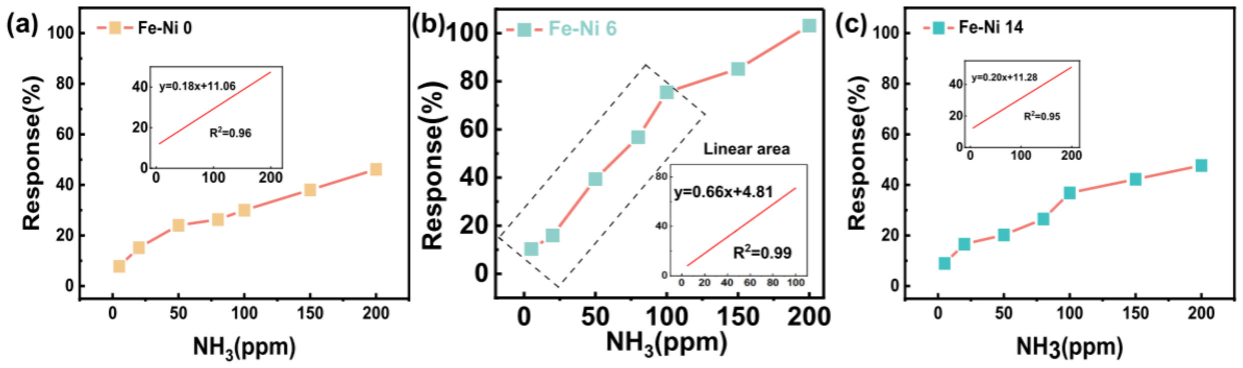

Figure S4. Linear relationship of response to different concentrations.

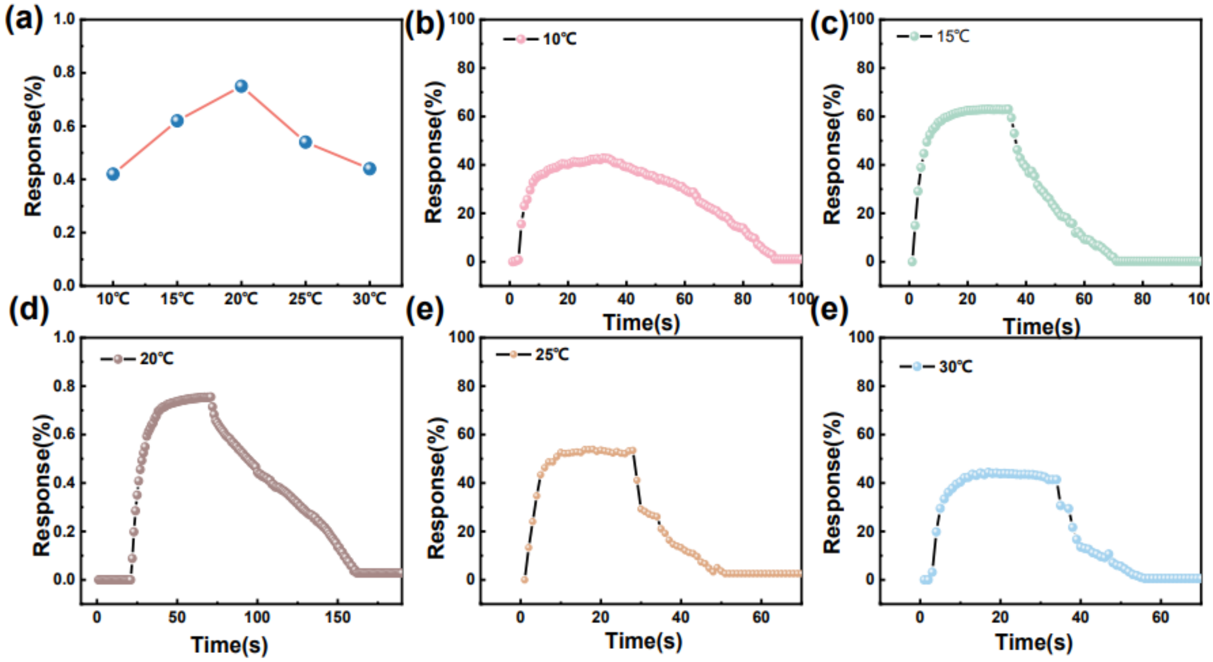

Figure S5. The different operating temperature of the response curve of Fe-Ni 6.

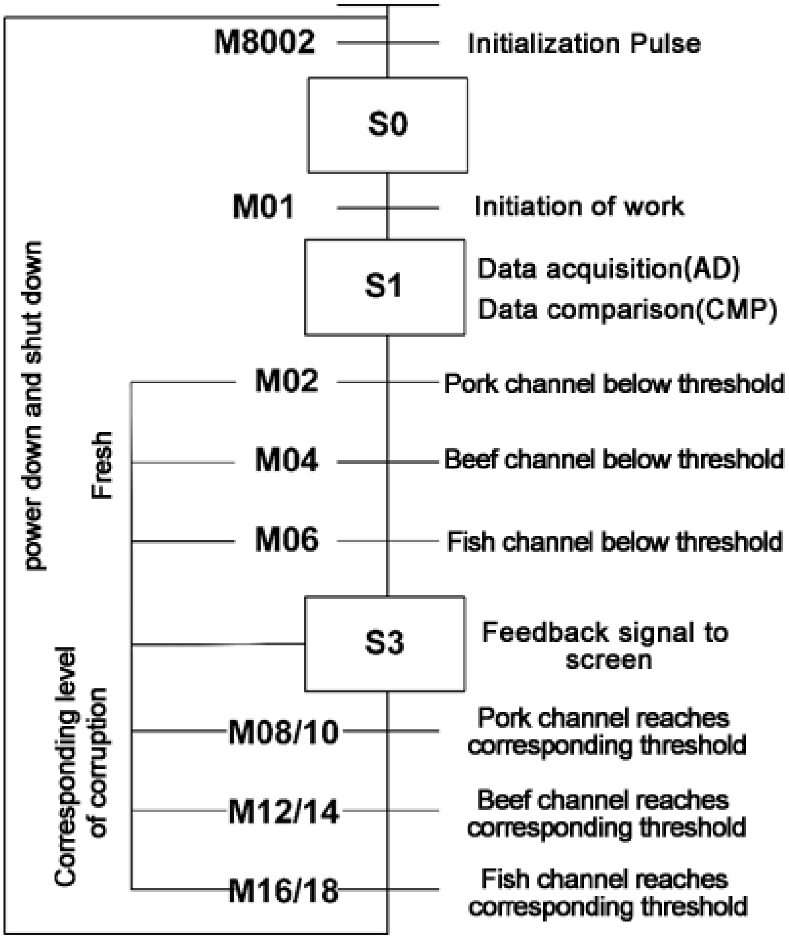

Figure S6. Principle of PLC control program.

		PH			Sensor value	
**Day**	**Pork**	**Beef**	**Fish**	**Pork**	**Beef**	**Fish**
0	5.54	5.43	6.7	0	0	0
0.25	5.68	5.53	6.5	2	1	1
0.5	5.7	5.66	6.64	8	9	6
0.75	5.82	5.71	6.72	10	12	8
1	5.9	5.8	6.8	12	15	10
1.25	6.13	5.83	6.82	21	18	14
1.5	6.27	5.83	6.86	24	27	17
1.75	6.35	5.88	6.89	31	29	19
2	6.4	5.9	7	39	34	21
2.25	6.43	6.15	7.04	41	38	24
2.5	6.48	6.26	7.21	41	40	27
2.75	6.5	6.48	7.36	43	43	31
3	6.53	6.5	7.5	45	45	32
3.25	6.59	6.58	7.59	48	47	41
3.5	6.62	6.78	7.65	53	52	46
3.75	6.65	6.89	7.68	57	57	51
4	6.67	7	7.7	59	62	54
4.25	6.66	7.01	7.72	62	64	59
4.5	6.68	7.08	7.76	69	66	67
4.75	6.7	7.17	7.77	71	68	72
5	6.71	7.2	7.8	75	69	74

Table S1. The results of the detection methods.

kind	fresh/response range（%）	primary corruption/response range（%）	total corruption/response range（%）
pork	0-24	24-48	≥48
beef	0-43	43-47	≥47
fish	0-10	10-21	≥21

Table S2. Threshold value of meat corruption detection system.
